# Impact Resistance Enhancement by Adding Core-Shell Particle to Epoxy Resin Modified with Hyperbranched Polymer

**DOI:** 10.3390/polym9120684

**Published:** 2017-12-07

**Authors:** Shuiping Li, Qisheng Wu, Huajun Zhu, Qing Lin, Chengshuang Wang

**Affiliations:** 1 School of Materials Science and Engineering, Yancheng Institute of Technology, Yancheng Jiangsu 224051, China; hjzhu2008@ycit.cn (H.Z.); wangcs@ycit.cn (C.W.); 2 Institute of Cement Science and New Building Materials, China Building Materials Academy, Beijing 10024, China; 3 School of Materials Engineering, Jinling Institute of Technology, Nanjing Jiangsu 211169, China; lnqing@jit.edu.cn

**Keywords:** epoxy, core-shell particle, hyperbranched polymer, impact resistance, reinforcement

## Abstract

A core-shell particle was fabricated by grafting amino-terminated hyperbranched polymer to the surface of silica nanoparticles. The influences of core-shell particle contents on the tensile and impact strength of the epoxy thermosets modified with amino-terminated hyperbranched polymer were discussed in detail. For comparison, core-shell particle was added into the epoxy/polyamide system for toughness improvement. Results from tensile and impact tests are provided. The introduction of core-shell particle into the epoxy/polyamide systems just slightly enhanced the tensile and impact strength. The incorporation of 3 wt % core-shell particle could substantially improve the tensile and impact strength of epoxy/amino-terminated hyperbranched polymer thermosets. Field emission-scanning electron microscope images of the impact fracture surfaces showed that the excellent impact resistance of epoxy/amino-terminated hyperbranched polymer/core-shell particle thermosets may be attributed to the synergistic effect of shearing deformation and crack pinning/propagation, which is induced by the good compatibility between epoxy matrix and core-shell particle in the presence of amino-terminated hyperbranched polymer.

## 1. Introduction

Epoxy resins are a class of high-performance materials and widely used as a mechanical material, structural adhesive, molding compound, electronic materials, functional coating and advanced composite matrix [1,2,3] owing to their excellent engineering properties. However, epoxy thermosets present poor impact resistance because of their high cross-linking density. For this reason, much attention has been paid to the modification of epoxy thermosets in recent years.

Besides modifying epoxy thermosets with rubbers, thermoplastic resins, and clay nanoparticles [4,5,6,7,8,9,10,11], it is usually considered that the addition of core-shell particles (CSPs) is an appropriate method to solve the poor impact resistance problem in epoxy resins [12,13,14,15,16,17]. CSPs are a kind of structure composite particles, which consist of at least two different components, such as core and shell parts [18]. Generally, the core part can be a solid, liquid or gas. However, the shell part is usually a solid, which may contain several terminal functional groups, depending on different applications [19]. CSPs with high flexibility property become more and more important for improving impact resistance of epoxy thermosets due to their easy processability and relative low cost [20]. However, the traditional CSPs, which consist of pure polymers as core and shell parts, do not exhibit desired effect when they are used to enhance the impact resistance of epoxy thermosets [20,21]. Therefore, many current studies have focused on the preparation of new CSPs with high impact property [13,16,22,23,24,25]. One novel approach is to incorporate inorganic nanoparticles into polymers to form inorganic/organic core-shell nanoparticles. These nanoparticles usually present excellent properties due to the synergistic effect of the unique mechanical performances from inorganic nanoparticles and varied functionality from organic polymers. Quan and Ivankovic presented that the fracture energy of epoxy resins could be increased from 343 to 2671 J/m^2^ by the addition of 30 vol % of CSR particles [21]. Thitsartarn and co-workers showed that the mechanical performances and toughness of epoxy composites could be simultaneously enhanced by the introduction of a novel inorganic/organic hybrid filler [20]. Wang et al. demonstrated that the epoxy resins with 5 wt % nanocomposites exhibited the optimal mechanical properties [26]. Zhong and Joshi suggested that the CSP particles significantly improved the impact properties of the GFRP laminates [27]. Zeng and co-workers supposed that the mechanical properties of composites increased with the increase of SiO_2_ thickness of CNT@SiO_2_ core-shell particles [28].

Hyperbranched polymers (HBPs), which show a high density of terminal functional groups, low melting point, and uniform three-dimensional architecture, are a fascinating kind of polymers [29]. There are several advantages when HBPs are used as modifiers in epoxy resins: (1) the compatibility of matrix and HBPs can be improved due to the high amount of terminal groups in the architecture of HBPs [30,31,32]; (2) a large number of free volumes and free spaces of cured networks can sharply improve the impact resistance of epoxy thermosets [33,34]; and (3) their unique sphere architectures can reduce the shrinkage of epoxy thermosets [34,35,36]. Zou and co-workers confirmed that the impact strength of the DGEBA/amine system with 10 wt % hyperbranched polyurethane was three times as large as the unmodified system [37]. Fei et al. introduced that the toughening and reinforcing mechanism of HBP for epoxy thermosets can be contributed to the synergistic effect of the architecture and increase of the cross-linking density and free volumes [38]. Misasi and co-workers suggested that the incorporation of the hybrid POSS-hyperbranched polymer in epoxy network resulted in a 220% increase in toughness owing to the excellent compatibility of the hyperbranched epoxy and POSS’ pendants [39].

To the authors’ knowledge, very few reports have described the preparation of core-shell particles consisting of amino-terminated hyperbranched polymer (ATHBP) as shell part and inorganic components as core part [40,41,42,43,44]. Our previous work demonstrated that the introduction of HBPs could improve the toughness of epoxy thermosets, while sacrificing the mechanical properties [30]. The toughness increment was short of expectation [31,45]. In the current work, our novel approach for the preparation of CSP is to grow ATHBP (the shell part) into the surface of silica nanoparticles (the core part). Then, the influence of CSP contents on the impact resistance of epoxy thermosets modified with ATHBP was discussed. For comparison, CSPs were also used to modify the epoxy/polyamide thermosets.

## 2. Experimental Section

### 2.1. Materials

The diglycidyl ether of bisphenol A (epoxy resin, E51) with an epoxide equivalent of 185–208 g/eq was purchased from Hangzhou Wuhuigang Adhesive Co., Ltd., Hangzhou, China. Polyamide 650 with an amine value of 220 ± 20 mg·KOH/g was supplied by Wuxi Resin Factory, Wuxi, China. Amino-terminated hyperbranched polymer (ATHBP) was synthesized according to our former work [45,46]. The silica nanoparticles with an average diameter of 60 nm were supplied by Yixing Xinxing Co., Ltd., Yixing, China. γ-Aminopropyl triethoxysilane was purchased from Sinopharm Chemical Reagent Co., Ltd., Shanghai, China. Aqueous solution of H_2_O_2_ (30 wt %), *N*,*N*-dimethylacetamide (DMA), *N,N*-dimethyl formamide (DMF), succinic anhydride, toluene, ethanol, diethylenetriamine (DETA) and diethanolamine were purchased from Chengdu Kelong Chemical Reagent Company, Chengdu, China. Unless otherwise mentioned, all chemical agents were analytical grade and used as-received.

### 2.2. Preparation of CSP

The preparation of CSP followed our previous work [30,32,45,46]. Typically, 1.4 g of silica nanoparticles was blended with 20 mL of H_2_O_2_ solution and sonicated at room temperature (RT) for 30 min in a 250 mL three-necked round-bottom flask, followed by refluxing at 105 °C for 4 h. The particles were washed with deionized water for three times, filtered and dried under vacuum at 80 °C for 12 h. These particles were named as hydroxylated silica. Then, the hydroxylated silica particles were added into 20 mL of toluene and sonicated at RT for 30 min in a 250 mL three-necked round-bottom flask. Five grams of γ-Aminopropyl triethoxysilane were added into the mixture and heated up to 80 °C for 24 h with magnetic stirring at 600 rpm under nitrogen flow. The resulting particles were washed with toluene and ethanol for three times, filtered and dried under vacuum at 80 °C for 12 h. These particles were named as silanized silica.

The above silanized silica particles were added into 5 mL of DMA and sonicated for 30 min at RT in a 50 mL three-necked round-bottom flask. Exactly 1.50 g of diethanolamine was added into the mixture and heated up to 70 °C under magnetic stirring at 600 rpm for 30 min. Then, 1.57 g of succinic anhydride was added and stirred at 300 rpm for another 30 min. After that, the mixture was heated up to 120 °C and stirred at 300 rpm for 6 h under nitrogen atmosphere. After the initial particles were cooled down to 110 °C, 3.1 g of DETA was added dropwise under nitrogen flow and stirred at 300 rpm for 10 h. The final particles (CSPs) were washed with DMA for three times and centrifuged at 5000 rpm for 10 min, and then dried under vacuum at 80 °C for 12 h.

### 2.3. Preparation of Epoxy/ATHBP/CSP Thermosets

For the preparation of epoxy/ATHBP/CSP thermosets, appropriate amounts of epoxy resin, CSP (CSP/epoxy, 0, 1, 2, 3, 4 and 5 wt %) and/or 10 wt % ATHBP were pre-heated at 60 °C for 5 min and the mixture was homogenized by mechanical stirring at 200 rpm for 20 min, followed by sonicated at 30 °C for 20 min. After that, appropriate amounts of polyamide 650 (polyamine/epoxy, 1.2:1 wt/wt), which was also pre-heated at 60 °C for 5 min, were added and the resulting mixture was also homogenized by mechanical stirring at 200 rpm for 10 min. Finally, the formulations were poured into a stainless steel template, which was also pre-heated at 60 °C for 5 min, and cured at 60 °C for 48 h in an oven.

### 2.4. Characterization

Attenuated total internal reflectance infrared spectroscopy (ATR-IR) spectra were collected on a Bruker Tensor 37 instrument through ATR-test (Bruker, Karlsruhe, Germany) within the range of 400–4000 cm^−1^ and a resolution of 4 cm^−1^. ^1^H nuclear magnetic resonance spectrum (^1^H NMR) was recorded on a Bruker AVANCE III 500 NMR spectrometer (Bruker, Zurich, Switzerland) with deuterated dimethyl sulfoxide (DMSO-d_6_) as the solvent at 293 K. Thermal gravimetric analysis (TGA) was measured using a DTG-60 simultaneous measuring instrument (SHIMADZU, Kyoto, Japan) from 30 to 800 °C at a heating rate of 20 °C·min^−1^. High resolution transmission electron microscope (HRTEM) was performed with a JEM-2100 high resolution transmission electron microscope (JEOL, Akishima, Japan) to observe the size and shape of core-shell particles. Tensile tests were performed on dumbbell-shaped specimens according to GB/T 2567-2008 with a SHIMADZU AG-X plus (SHIMADZU, Kyoto, Japan) test machine with a loading rate of 2 mm/min. The tensile sample size was 200 mm × 10 mm × 4 mm. Un-notched impact strength tests were performed with a ZBC 50 pendulum impact testing machine (New SANS, Shenzhen, China) without a notch in the specimen according to GB/T 2567-2008 standards. The impact sample size was 80 × 10 × 4 mm^3^. Five specimens were tested and averaged to determine the tensile and impact strength. After impact testing, field emission scanning electron microscopy (FESEM) images were recorded using a Hitachi SU8000 field-emission scanning electron microscope (Hitachi, Tokyo, Japan), and the fracture surfaces of the specimens were sputter-coated with gold before observation.

## 3. Results and Discussion

### 3.1. Characterization of CSPs

**Figure 1:**
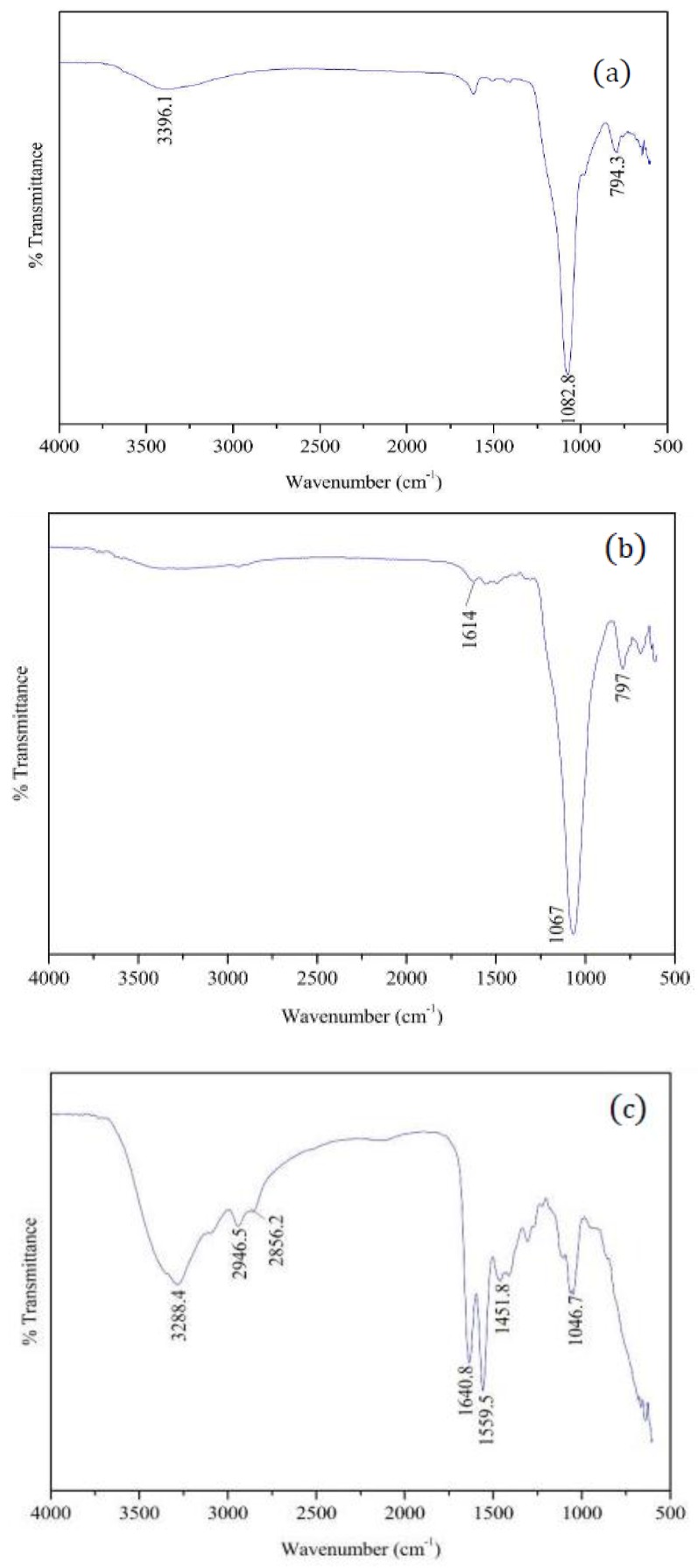
The ATR-IR spectra of: hydroxylated silica (**a**); silanized silica (**b**); and core-shell particle (CSP) (**c**) particles.

The ATR-IR spectra of hydroxylated silica, silanized silica and CSP particles are shown in Figure 1. The weak peak at 794 cm^−1^ and sharp peak at 1083 cm^−1^ in the ATR-IR spectrum of hydroxylated silica can be assigned to Si–O and Si–O–Si stretching vibration, respectively. The broad absorption at 3396 cm^−1^ is attributed to hydroxyl group (–OH) stretching. The spectrum of silanized silica particles is similar to that of hydroxylated silica, but two differences can still be observed. On the one hand, the weak peak appeared at 1614 cm^−1^ can be ascribed to N–H stretching. On the other hand, peak at 3396 cm^−1^ disappeared. These two differences may indicate that the hydroxylated silica particles are successful silanized by γ-APS. In the spectrum of CSPs, the broad peak at 3288.4 cm^−1^ is assigned to the N–H stretching vibration of the primary amine. The peaks at 2856.2 and 2946.5 cm^−1^ can be attributed to the symmetry and asymmetry stretching vibration of C–H, respectively. The peak at 1640.8 cm^−1^ is assigned to –CONH– stretching vibration. The peak at 1559.5 cm^−1^ is attributed to N–H bending vibration. The peak at 1451.8 and 1046.7 cm^−1^ can be assigned to C–N bending and stretching vibration, respectively. These results indicated that CSPs have been successfully fabricated through grafting ATHBP to the surface of silanized silica particles.

**Figure 2:**
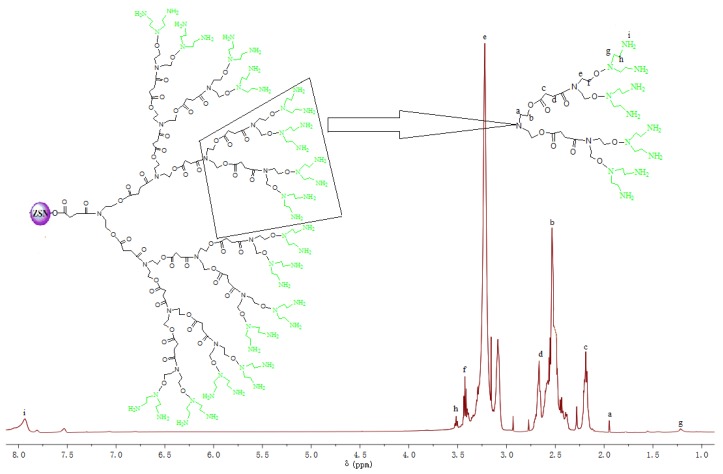
^1^H NMR spectrum of CSPs.

The ^1^H NMR spectrum of CSPs provides more detailed evidence to prove the successful growth of ATHBP in the surface of silanized silica particles (Figure 2). The ^1^H NMR spectrum clearly displays the characteristic peaks of ATHBP. The peaks at 1.779 (protons a), 2.533 (protons b), 2.188 (protons c) and 2.665 (protons d) ppm are assigned to the linear units. The peaks at 3.222 (protons e) and 3.414 (protons f) ppm are ascribed to the dendritic units. The peaks at 1.554 (protons g), 3.503 (protons h) and 7.82 (protons i) ppm are attributed to the terminal units.

**Figure 3:**
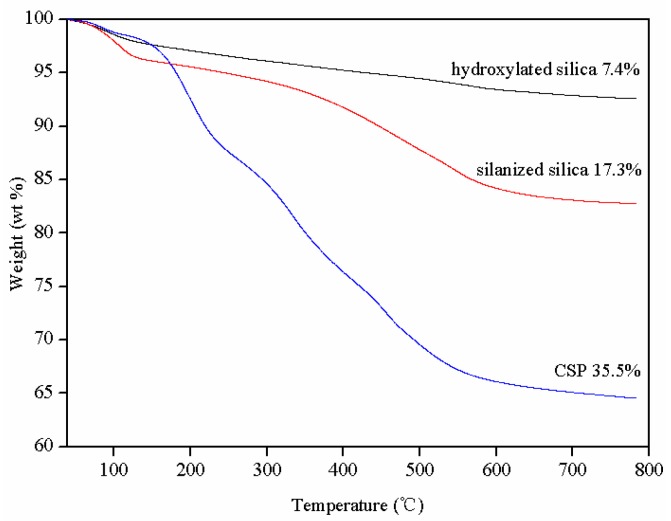
TGA curves of hydroxylated silica, silanized silica and CSPs measured under N_2_.

The TGA curves of hydroxylated silica, silanized silica, and CSP particles measured under N_2_ atmosphere are shown in Figure 3. As can be seen, the final weight losses of hydroxylated silica, silanized silica, and CSPs are 7.4%, 17.3% and 35.5% at 800 °C, respectively. Obviously, the weight losses of these particles follow the order of hydroxylated silica < silanized silica < CSPs at 800 °C. This may be a possible evidence to approve the successful grown of hydroxyl, amino group and ATHBP into the surface of silica nanoparticles, respectively. Moreover, the temperatures for 5 and 10 wt % mass loss from the CSPs are 177 and 213 °C, respectively, which may indicate a good thermal stability of CPSs. It can be calculated that the grafting rate of ATHBP to the surfaces of CSPs as shell part is 18.2 wt %, which may result in good compatibility between CSPs and epoxy matrix in the presence of ATHBP.

**Figure 4:**
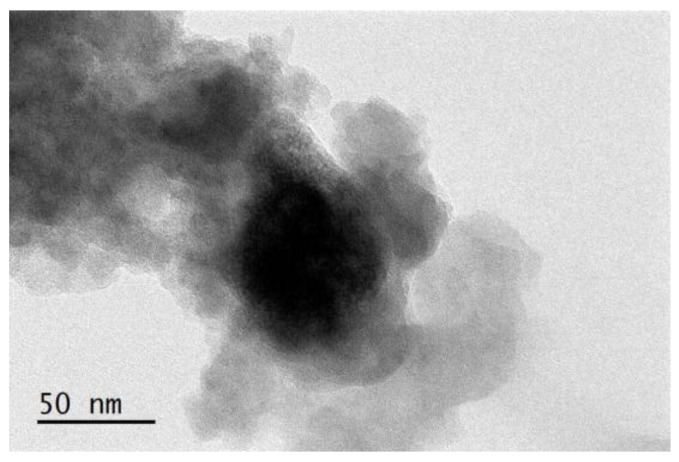
HRTEM morphology of CSPs.

The HRTEM morphology of CSPs, as shown in Figure 4, helps illustrate the size, shape and dispersion status of the particles. It can be observed that the clusters slightly aggregate owing to their high density terminal groups, huge specific surface areas and high surface energy. The particle sizes of CSPs range from 60 to 80 nm. A thick layer is obviously present on the surface of CSPs. Based on the above analysis, it can be concluded that ATHBP has been grafted to the surface of silanized silica particles, thus CPSs were successfully prepared.

### 3.2. Mechanical Properties of the Epoxy Thermosets Modified with CSPs

**Figure 5:**
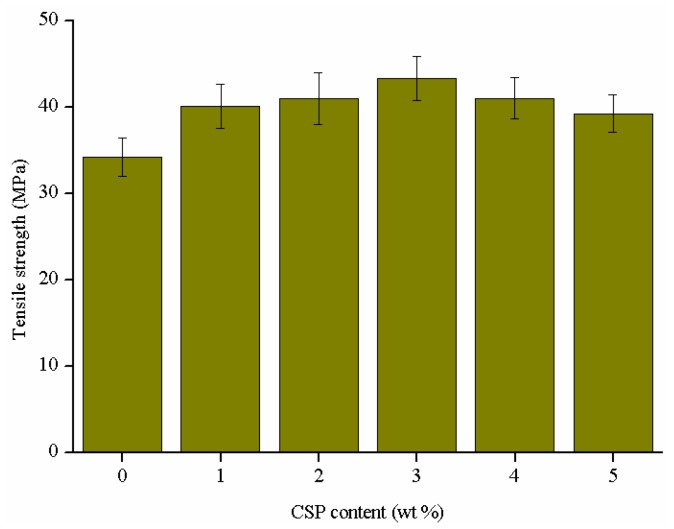
Tensile strength of the epoxy thermosets modified with different contents of CSPs.

Tensile and impact strength are two important indexes for evaluating the mechanical properties of the epoxy thermosets. The tensile strength of the epoxy thermosets modified with different contents of CSPs is presented in Figure 5. The tensile strength increases steadily from 34.23 MPa for the unmodified epoxy thermosets to 43.33 MPa for the formulation with 3 wt % CSPs (about 26.6%), after that the trend alters and decreases to 39.22 MPa for the formulation with 5 wt % CSPs. Obviously, the enhancement of the tensile strength can be attributed to the interaction between CSPs and epoxy matrix, which can strengthen the interfacial adhesion and result in the improvement of tensile failure resistance.

**Figure 6:**
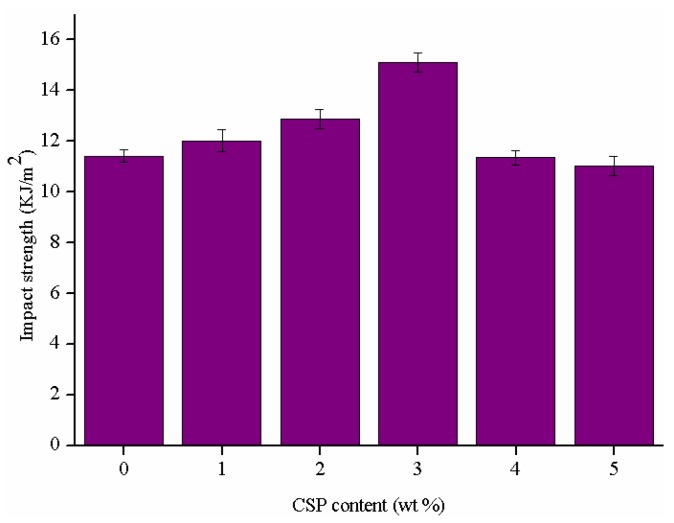
Impact strength of the epoxy thermosets modified with different contents of CSPs.

The impact strength of the epoxy thermosets modified with different contents of CSPs is shown in Figure 6. The impact strength of CSPs modified epoxy thermosets increases by 5.3%, 12.7%, 32.2%, −0.03% and −0.6%, respectively, in relation with the unmodified epoxy thermosets. The good compatibility and strong interaction between epoxy matrix and functional groups of CSPs are contributed to the improvement of the impact strength of the formulations modified with 1, 2 and 3 wt % CSPs. The improvement may be attributed to a stress transfer effect, the initiation of micro-crack and crack pinning/bridging effect of the CSPs.

It can be noted that the tensile and impact strength decrease with increasing the CSPs content when the CSPs content is higher than 3 wt %. This may be due to the aggregate of CSPs, which results in reducing the interfacial adhesion between CSP particles and polymer matrix. Moreover, the aggregation can also induce stress concentration and lead to the generation of macro-cracks [47]. Overall, the tensile and impact strength of the formulation with 3 wt % CSPs were increased by about 26.6% and 32.2%, respectively.

### 3.3. Mechanical Properties of the Epoxy/ATHBP Thermosets Modified with CSPs

**Figure 7:**
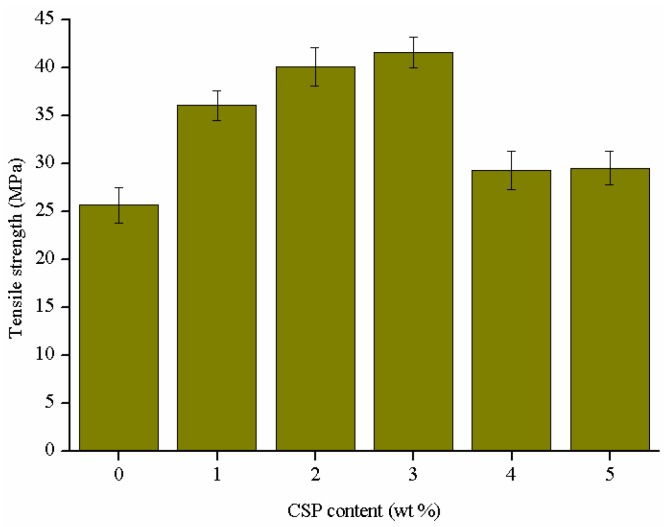
Tensile strength of the epoxy/amino-terminated hyperbranched polymer (ATHBP) thermosets modified with different contents of CSPs.

It is well known that HBP is an excellent kind of tougheners for epoxy thermosets or composites [30,31]. However, the introduction of HBPs may affect the tensile strength and other mechanical performances. CSPs are used as nanofillers to improve the mechanical behaviors of the epoxy thermosets modified with a certain amount of ATHBP. Figure 7 shows the tensile strength of the epoxy thermosets that contain 10 wt % ATHBP and modified with different contents of CSPs. The tensile strength is greatly dependent on the CSPs content and higher than that of the epoxy thermosets with 10 wt % ATHBP. For instance, the tensile strength of the formulation with 3 wt % CSPs increases by about 61.9%, compared to the epoxy thermosets with 10 wt % ATHBP. The increment may be attributed to the strong interaction between epoxy matrix and ATHBP, which is grown on the surface of silica nanoparticles. The decrease can also be attributed to the aggregation of CSPs when the content is higher than a threshold.

**Figure 8:**
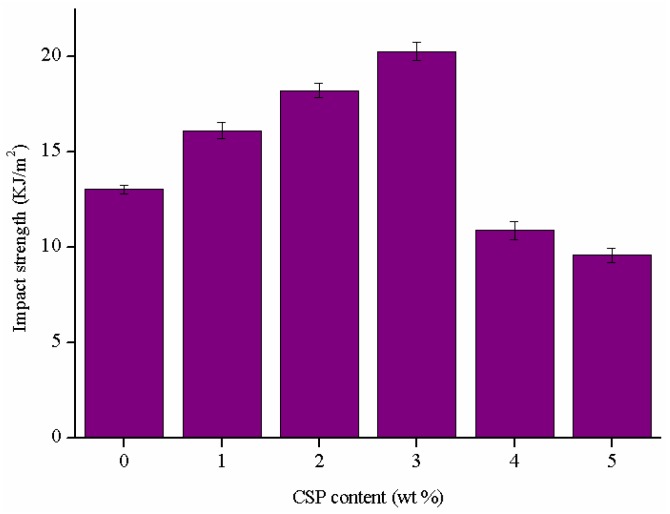
Impact strength of the epoxy/ATHBP thermosets modified with different contents of CSPs.

The impact strength of the epoxy thermosets that contains 10 wt % ATHBP and modified with different contents of CSPs are shown in Figure 8. The impact strength of the formulation with 3 wt % CSPs increases by about 55.5%, in relation with the unmodified epoxy/ATHBP thermosets. Many terminal functional amino groups on the surfaces of CSPs may contribute to enhancing the compatibility between CSPs and epoxy matrix, thus improving the impact resistance. The presence of the CSPs also deflects the crack, increases the fracture surface area, and results in increasing the fracture energy of the modified thermosets [11].

The above results indicated that the addition of a small amount of CSPs in epoxy/ATHBP thermosets can great enhance the tensile and impact strength. Similarly, the decrease of the impact strength may be attributed to the presence of the aggregation of CSPs, which is owing to the high surface energy, particle concentrations and/or inefficient dispersion [48].

### 3.4. Fracture Surface Morphologies

**Figure 9:**
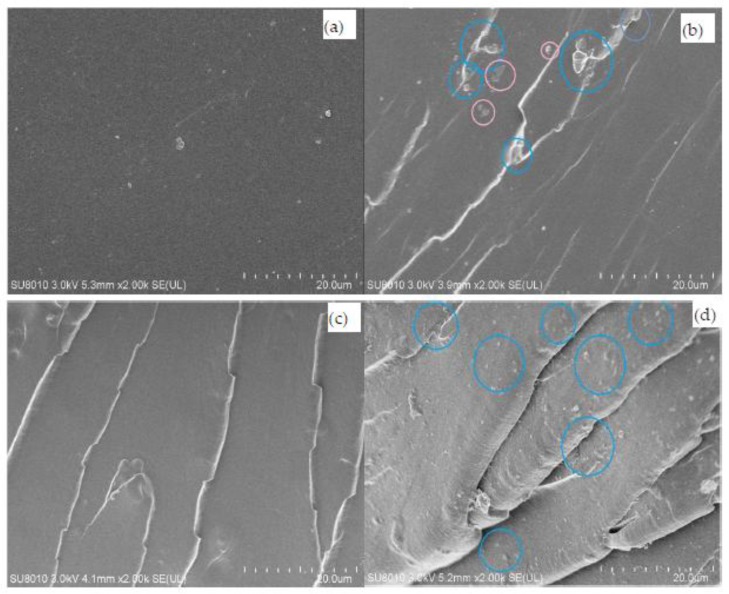
FESEM images of: the unmodified epoxy thermosets (**a**); the epoxy thermosets modified with 3% CSPs (**b**); the epoxy thermosets with 10 wt % ATHBP (**c**); and the epoxy/ATHBP thermosets modified with 3% CSPs (**d**).

The impact resistance improvement of the epoxy thermosets modified with CSPs can be explained in terms of the impact fracture surface morphologies observed by FESEM. The fracture surface after impact resistance tests was investigated by this technique. Figure 9 shows the FESEM images for the impact fracture surfaces of the unmodified epoxy and epoxy/ATHBP thermosets modified with different contents of CSPs. It is easy to observe that all the surface micrographs present a homogeneous appearance, which indicates that there is no phase separation in the epoxy thermosets and accounting for a good compatibility. Figure 9a shows the impact fracture surface micrograph of the unmodified epoxy thermosets. It is evident that the fracture surface is relatively smooth and glassy, showing a typical morphology of brittle polymer thermosets and resulting in poor resistance to crack initiation and uninterrupted crack propagation path under impacting. Figure 9b depicts the fracture surface morphology of the epoxy thermosets modified with 3 wt % CSPs and exhibits a rough fracture. A part of the CSPs are nailed in the front of cracks and hinder the crack propagation, and thus the impact resistance can be improved. Other part of the CSPs are protruded clearly in the fracture surface, which indicates that the interface of epoxy matrix and CSPs is the weaknesses in the thermosets. The impact fracture surface image of the epoxy thermosets with 10 wt % ATHBP (Figure 9c) also shows a rougher surface, higher amounts of crack and more shearing deformation than that of the unmodified epoxy thermosets. Compared with the morphology of the unmodified epoxy thermosets, the crack propagation directions are scattered. It is well known that when cracks occur, the shearing deformation can absorb impact fracture energy [49] and interrupt crack propagation. This well-known mechanism indicates that the impact fracture energy of the epoxy thermosets modified with 10 wt % ATHBP can be increased due to the presence of the shearing deformation and crack propagation. CSPs shows good dispersion and homogeneity in the fracture surface morphology of the epoxy/ATHBP thermosets modified with 3 wt % CSPs (Figure 9d). The uniform distribution of CSPs in the thermosets can be attributed to the strong interaction between epoxy matrix and particles [50], which is due to a large amount of the terminal functional groups in the surfaces of CSPs. Moreover, CSPs were coated with epoxy matrix and then embedded in the matrix.

### 3.5. Mechanism

According to other researchers’ reports [29,51,52], phase separation usually occurs when HBPs are used as non-reactive modifiers, and the toughening mechanism can be explained similar to that of the rubber toughening [51]. However, no phase separation was observed in our work. Thus, the impact resistance mechanism can be explained as follows.

Firstly, the addition of ATHBP can introduce a high amount of free volumes and free spaces in epoxy networks [33,34,53], which can increase shearing deformation and thus improve the impact resistance [29]. Secondly, the high density of terminal functional groups in the architecture of ATHBP can enhance the compatibility between ATHBP and epoxy matrix [30,31,32]. Thirdly, the incorporation of CSPs can deflect the crack and thus enhance the fracture energy and dissipate more plastic energy of the thermosets [32]. Finally, many terminal functional groups in the shell of CSPs can improve the compatibility and strengthen the interface of CSPs and epoxy matrix. Thus, it can be concluded that the mechanism of impact resistance improvement can be attributed to the synergistic effect of the shearing deformation and crack pinning/propagation, which are owing to the introduction of ATHBP and CSPs, respectively.

## 4. Conclusions

A core-shell particle (CSP) was fabricated by grafting an amino-terminated hyperbranched polymer (ATHBP) to the surface of silica nanoparticles. The influence of CSP contents on the impact resistance of the epoxy thermosets modified with ATHBP was investigated. The results indicated that the tensile and impact strength of the epoxy thermosets were dependent on the contents of CSPs. The addition of an appropriate amount of CSPs can favorable improve the impact strength of the epoxy thermosets owing to the crack pinning. The tensile and impact strength of the epoxy/ATHBP thermosets could be greatly enhanced by the addition of 3 wt % CSPs. The impact resistance improvement can be attributed to the shearing deformation induced by the introduction of ATHBP, which contains many terminal functional groups, and the crack pinning/propagation caused by the addition of CSPs, which exhibit good compatibility with epoxy matrix in the presence of ATHBP.
